# The premammillary nucleus of the hypothalamus is not necessary for photoperiodic timekeeping in female turkeys (*Meleagris gallopavo*)

**DOI:** 10.1371/journal.pone.0190274

**Published:** 2018-02-20

**Authors:** Ashli F. Moore, Vincent M. Cassone, Kevin D. Alloway, Paul A. Bartell

**Affiliations:** 1 Department of Biology, University of Kentucky, Lexington, Kentucky, United States of America; 2 Department of Neural and Behavioral Sciences, Pennsylvania State University College of Medicine, Hershey, Pennsylvania, United States of America; 3 Department of Animal Science, Pennsylvania State University, University Park, Pennsylvania, United States of America; Karlsruhe Institute of Technology, GERMANY

## Abstract

In birds, seasonal reproduction is regulated by day length, with long days in the spring activating the hypothalamic-pituitary-gonadal axis and reproductive behaviors. The photoreceptors mediating this process remain unknown, but recently, the premammillary nucleus (PMM) of the hypothalamus has been implicated as the site of photoperiodic signaling in turkeys. We performed electrolytic lesions of the PMM to elucidate its role in the photoactivation and maintenance of egg production in female turkeys. Our results show that ablation of the PMM does not alter the normal lay cycle. No differences were found between lesioned birds and sham controls in the latency to lay following photostimulation, nor in subsequent egg production over a period of 29 weeks. No differences in the incidence of gonadal regression were found, indicating that the PMM is not essential for the termination of breeding. We conclude that any role of the PMM in photoperiodic regulation, if it exists, is redundant with other components of the system.

## Introduction

Many avian species breed seasonally, using the annual changes in day length to synchronize reproduction with favorable environmental conditions. Under short-day conditions in winter, most birds are reproductively quiescent and the gonads are in a regressed state. Increasing day length in the spring stimulates the reproductive axis, leading to gonadal growth and reproduction. After continued exposure to long photoperiods, reproductive activity ceases, the gonads regress, and the birds become photorefractory (insensitive to the stimulating effects of long days). Photorefractoriness ensures that reproduction does not occur during unfavorable conditions in winter. After exposure to short days, the animal regains photosensitivity and the cycle starts over. This process is known as photoperiodism (reviewed in [1]). The photoperiodic system requires a photoreceptor to detect the light signal, a circadian clock to measure day length, and an output pathway coupled to the reproductive system [2]. It is important to note that, unlike rodent species which show spontaneous gonadal recrudescence under prolonged short photoperiods [3], most avian species, including the domestic turkey, require photostimulation for gonadal development [4].

In birds, it is well-established that neither the eyes nor the photosensitive pineal gland are required for the perception of photoperiodic information [5, 6]. Rather, the photoreceptors involved in photoperiodic timekeeping are located within the brain, in or near the mediobasal hypothalamus (MBH) [2]. These deep-brain photoreceptors (DBPs) detect light signals and—if the photoperiodic circadian clock determines that light is occurring late in the day—trigger a cascade of neuroendocrine events leading to the activation of the gonads. The neuroendocrine control of photoinduction has been elegantly illustrated by studies in Japanese quail (*Coturnix japonica*). Long days induce the expression of thyroid stimulating hormone (TSH) in the pars tuberalis (PT) of the pituitary, which upregulates the expression of the gene for Type 2 deiodinase (*Dio2*) in the MBH. Dio2 enzyme converts thyroid prohormone (T_4_) to its more active form (T_3_) [7–9]. It appears that increased T_3_ in the MBH promotes the secretion of gonadotropin releasing hormone (GnRH) from GnRH neuron terminals in the median eminence (ME) by altering neuroglial interactions [10, 11]. GnRH stimulates the release of gonadotropins (luteinizing hormone, LH, and follicle stimulating hormone, FSH) from the pituitary, which enter the circulation and promote gonadal growth and reproduction [12]. In contrast, photorefractoriness is associated with decreased GnRH secretion after prolonged exposure to stimulating photoperiods, resulting in sustained gonadal regression [12, 13].

Although the neural mechanisms of photoinduction have been well-described, the exact location of the DBPs remain elusive. Recent works characterizing opsin expression in the avian brain have uncovered 4 candidate locations, expressing several distinct opsins, that may serve as DBPs. 1) In chickens (*Gallus domesticus*), lesioning the lateral septal region, which expresses the circadian photopigment melanopsin (OPN4), attenuates photostimulation of the gonads [14, 15], and electrophysiological data suggest that cells in the lateral septal organ (LSO) are intrinsically photosensitive [16]. 2) Electrophysiological studies in Japanese quail show that cerebrospinal fluid-contacting neurons in the paraventricular organ (PVO), which express neuropsin (OPN5) and appear to send projections to the ME, are intrinsically photosensitive [11, 17]. 3) In chicken and Japanese quail, the paraventricular nucleus (PVN), a structure known to be involved in the mammalian photoperiodic signaling pathway [18, 19], expresses vertebrate-ancient opsin in the perikarya as well as in fibers projecting to the ME [20]. 4) The premammillary nucleus (PMM) of turkeys (*Meleagris gallopavo*), which also expresses melanopsin [21], shows patterns of clock gene expression suggesting the presence of a circadian clock that responds to changes in photoperiod [22] and is putatively coupled to the hypothalamic-pituitary-gonadal (HPG) axis via axonal and/or neurochemical pathways [21, 23]. Among these candidates, it remains to be established which of these sites not only detects photoperiodic light signals, but also is essential for photic control of gonadal growth.

The present study focuses on the PMM, a small area of the caudal MBH that has been implicated as the site regulating seasonal reproduction in turkeys. Light given during the photoinducible phase (when light signals are interpreted as a long-day stimulus) activates neurons in the PMM, as measured by changes in *c-fos* mRNA [24]. This activity coincides with light-induced activation of GnRH neurons and upregulation of *GnRH-1* mRNA in the bed nucleus of the pallial commissure (nCPa) [24, 25], a response that is attenuated by PMM lesions [26]. In addition, melanopsin is rhythmically expressed in the turkey PMM, with peak expression during the photosensitive phase [21]. Circadian rhythms in clock gene expression in the PMM differ under short and long photoperiods and are altered by a light pulse during the photoinducible phase, suggesting that a circadian clock in the PMM is responsive to photoperiodic information [22]. Furthermore, this circadian oscillator appears to drive an output system with close ties to the reproductive system [21, 22]. Some neurons in the PMM are immunoreactive to tyrosine hydroxylase (TH), the rate limiting enzyme in dopamine biosynthesis, as well as tryptophan hydroxylase 1 (TPH1), the first enzyme in the melatonin biosynthesis pathway, suggesting that these cells may produce both dopamine and melatonin [27]. Dopaminergic signaling in the hypothalamus is associated with photoperiodic regulation of avian reproduction [27–29], and melatonin signaling plays a key role in photoperiodism in mammals (although in birds, melatonin does not appear to be essential for photoperiodic activation of reproduction) [1, 13, 30]. In the turkey PMM, both *TH* and *TPH1* mRNA cycle rhythmically in constant darkness (in opposing phases), indicating that both transcripts are driven by an endogenous circadian clock, and their expression patterns differ under short- and long-day conditions [27]. TH-immunoreactive neurons originating in the PMM have been found in association with TSH neurons in the PT and anterior pituitary, raising the hypothesis that the PMM conveys photoperiod information to the reproductive axis via dopaminergic signaling [21].

Taken together, these findings suggest that the turkey PMM may contain a light-sensitive circadian oscillator, comprised of melanopsin photoreceptors and canonical clock gene machinery, coupled to dopamine and melatonin output. This system is sensitive to changes in photoperiod, and may integrate this information with the HPG axis to regulate gonadotropin release and the reproductive cycle [23]. Despite this line of evidence, none of these studies established that the PMM is necessary for reproductive output. Therefore, we ablated the PMM to determine its functional role in regulating photoperiodic reproduction. Specifically, we tested the hypotheses that the PMM mediates photoinduction, maintenance, and/or termination of egg production in female turkeys.

## Methods

### Housing

Turkey poults (Nicholas 500, Aviagen Turkeys, Lewisburg, WV) were obtained on hatch day and housed under standard breeder housing conditions until 26 weeks of age. Poults were initially kept under 24 hours of constant light (incandescent overhead lighting) and infrared brooding lamps. At day 2 of age, overhead lights were changed to a 14L:10D schedule. Starting at one week of age, heat was gradually reduced over 9 weeks from ~29°C to a target temperature of 20–21°C and infrared brooding lamps were removed. Room lighting intensity measured at the level of the birds' heads was maintained at 10 lux for the remainder of the study. This light intensity was used to inhibit aggression and resulted in egg production that met performance targets for the strain [31]. Food and water were provided *ad libitum*; custom-formulated starter and grower diets (Wenger Feeds, Rheems, PA) were used as appropriate. Lighting conditions were changed to 8L:16D at 16 weeks of age to condition the birds for photostimulation. Room doors were light-proofed and light baffles were placed over ventilation fans to ensure the integrity of dark conditions. All procedures were approved by the Institutional Animal Care and Use Committees at Pennsylvania State University and the University of Kentucky (approval #s 43762 and 2013–1124, respectively).

### Experimental groups

Birds were randomly assigned to one of two groups: birds in the unstimulated (U) group (N = 40) were kept under short-day conditions (8L:16D) and remained unstimulated until after surgery, whereas birds in the stimulated (S) group (N = 42) were stimulated by light (16L:8D) prior to surgery. After surgery, all birds were placed under photostimulating (16L:8D) conditions, where they remained for the duration of the study. The U group allows us to test the hypothesis that the PMM is involved in photostimulation, whereas the S group reveals the role of the PMM in the maintenance and/or cessation of egg-laying following normal photostimulation. Groups of ~4 birds were transferred to photostimulating conditions each day, starting at 26 weeks of age, to create the S group. Pubic bone spacing, squatting behavior, and egg-laying were monitored in photostimulated S group birds prior to surgery. Surgery was performed when photostimulation was confirmed, i.e., within 0–2 days of egg-laying. Concurrently, we also began surgeries on birds in the U group, alternating surgeries between the S and U groups. The resulting age-matched birds ranged between 28–33 weeks (surgery) and 26–32 weeks (photostimulation).

### Surgery

Bilateral electrolytic lesions of the PMM were performed under general anesthesia. Food and water were withheld 2 hours prior to surgery. To reduce bleeding and cerebral edema during and after surgery, birds were injected with dexamethasone (1 mg/kg) 20 minutes prior to anesthetic induction. Anesthesia was induced and maintained with isoflurane (3.5–5% induction; 2–3.5% maintenance), delivered via a nose cone for induction, and via tubing inserted in the mouth and nares for maintenance. Anesthetic depth was monitored by examining muscle tone, toe pinch reflex, corneal reflex, and respiratory rate. Anesthetized birds were placed into a stereotaxic instrument (David Kopf, modified for turkeys using a pigeon bite plate and 45° slide adaptor) with the head positioned at a 45° angle. Coordinates for the PMM lesion were 0.0 mm lateral-medial, +10.6 mm rostral-caudal, and +1.6 mm dorsal-ventral, relative to the reference (interaural zero).

An incision was made through the skin on the top of the head to expose the skull. A surgical drill was used to make a small hole (~1.5mm diameter) in the skull over the target coordinates, drilling ventrally until the dura mater was visible. A modified sterile needle was used to pierce the dura. An electrode (stainless steel insect pins, size #7, insulated, with 0.5 mm tip exposed) was lowered into the brain according to target dorsoventral coordinates. To make the lesion, positive current (1mA) was applied for 15 seconds using a DC constant current lesion maker (Grass Instruments Model D.C. LM5). Midline lesions with this protocol resulted in bilateral ablation of the PMM. The electrode was allowed to cool for 2 minutes before being withdrawn. Sterile gel foam was applied to the craniotomy and the skin was sutured shut. The bird was monitored closely for ~1 hr and then returned to experimental housing, where it was kept in a separate recovery pen for ~18–24 hrs before being released into the open room with the other birds. Within each experimental group (U and S groups), a subset of birds received sham lesions in which the electrode was lowered into the brain, but current was not applied.

### Monitoring egg production

Following surgery, birds were placed into one of two identical light-tight rooms (33.5 m^2^) with photostimulating lighting (16L:8D; 10 lux at bird head level, incandescent overhead bulbs). Birds from U and S groups were evenly distributed between the two rooms. Food and water were available *ad libitum*; a custom-formulated breeder diet (Wenger Feeds, Rheems, PA) was introduced following photostimulation and used for the remainder of the study. To monitor egg-laying in individual birds, each room was equipped with 16 custom-built trap nest boxes (71H X 76D X 61W cm). Upon entering the nest box, the trap door closed, preventing entry by other birds and prohibiting the trapped bird from leaving. Every 2–3 hours, nest boxes were checked: we recorded bird ID and egg presence (yes/no), removed any eggs, removed trapped birds, and re-set the trap doors. Check times were ZT0, 3, 6, 8, 10, 12, 15 (ZT = *zeitgeber time*, i.e., hours after lights-on). Forced ejection from nest boxes is known to promote continued egg-laying while inhibiting incubation behaviors and is routinely utilized in commercial operations to maximize production. In our study, it allowed us to quantify egg production in individual birds. All birds received at least 12 weeks of continuous trap nesting following photostimulation, capturing the onset of lay and peak production in all birds. Afterwards, birds were given access to nest boxes without traps for 4 weeks (eggs were removed from the room daily at ZT0). Thereafter, we intermittently monitored individual egg production by trap nesting one out of every three weeks for the next 13 weeks (a total of 5 trap nesting periods during the latter part of the laying cycle).

### Histology

At the conclusion of the experiment, birds were sacrificed via decapitation following electrostunning. Ovaries were inspected and classified as either regressed (reduced/atretic follicles) or active (hierarchical follicles). Brains were removed, flash-frozen in 2-methylbutane cooled with dry ice, and stored at -80°C. Brains were cryosectioned (20 μm), thaw-mounted onto gelatin-coated slides (two alternating sets were collected), and post-fixed in 4% paraformaldehyde. One set of slides was Nissl stained with thionin to determine the accuracy and extent of the lesions. As others have done [25], we first identified PMM cells using immunohistochemistry for TH; comparison with thionin-stained slides showed that PMM cells could be readily identified with thionin stain due to their distinct size, shape, and location. Lesion categories were independently verified by two observers. "Full lesions" were those in which no PMM cells remained; "partial lesions" were those in which damage could be detected, but PMM cells remained; "missed lesions" were those in which it was clear that the entire PMM remained intact. We also verified that sham-lesioned animals did not sustain PMM damage.

### Data analysis

Individual egg data were recorded in a binary format (egg = 1 or 0) according to date. Because photostimulation occurred on different dates across individuals, date was converted into "number of days following photostimulation." Lesions that missed the PMM were grouped together with shams. Within each photostimulation group (U and S), the explanatory variable was lesion category: sham/missed lesions, partial lesions, and full lesions. We analyzed the following dependent variables: 1) "latency," the number of days following photostimulation until the first egg was laid, 2) "total12," total egg production during the first 12 weeks following photostimulation, 3), "eggs/wk, " mean weekly egg production for the duration of the study, and 4) "egg production curves," individual weekly egg production over the duration of the study. All analyses were carried out in SAS (SAS Institute Inc., Cary, NC; version 9.4). We tested all dependent variables for homogeneity of variance and normality of distribution. Variance was homogenous for all variables, but none of the variables were normally distributed. For the simple count data (latency and total12), we tested goodness-of-fit for Poisson and negative binomial distributions using the Pearson’s χ^2^ goodness-of-fit test, and negative binomial was found to be a good fit for both variables (*p* = 0.22 for latency, *p* = 0.83 for total12). Therefore, we used statistical procedures for which this distribution could be specified, or that did not make assumptions about the distribution of the data. For dependent variables latency, total12, and eggs/wk, we used the Kruskal-Wallis test. In addition, for latency and total12, we performed negative binomial regression with a generalized linear model, fit with the GENMOD procedure in SAS. A Type III test was used to evaluate the overall effect of lesion category. The fourth variable, egg production curves, involves repeated sampling within individuals (weekly egg counts for each week of trap nesting for each individual) with missing data across time (weeks without trap nesting) that varies among individuals according to photostimulation date. To assess whether or not lesions had an effect on egg production curves, we utilized the GLIMMIX procedure in SAS to fit a generalized linear mixed model to these data. A negative binomial distribution was found to be a good fit for these data (*p* = 0.923, Pearson’s χ^2^ goodness-of-fit test), so this distribution was specified for the dependent variable to obtain a negative binomial regression. We modeled the fixed effects of lesion category, time (in weeks), and their interaction; in addition, since the egg curve is not linear, we also modeled time as a 2^nd^, 3^rd^, and 4^th^ order polynomial. Repeated sampling for individuals over time was specified as a random residual effect. We used a spatial power structure for the covariance in the repeated-measures effect, since the time intervals of egg sampling were not evenly spaced across the entire study. Hypothesis tests for the significance of main effects were evaluated with a Type III test of fixed effects. Marginal means for lesion category, adjusted for other model effects, were generated using the LSMEANS option; differences between the control group (sham/missed) vs. partial and full lesions were evaluated. The incidence of gonadal regression was compared among lesion categories using Fisher’s exact test.

## Results

### Lesions

**Fig 1:**
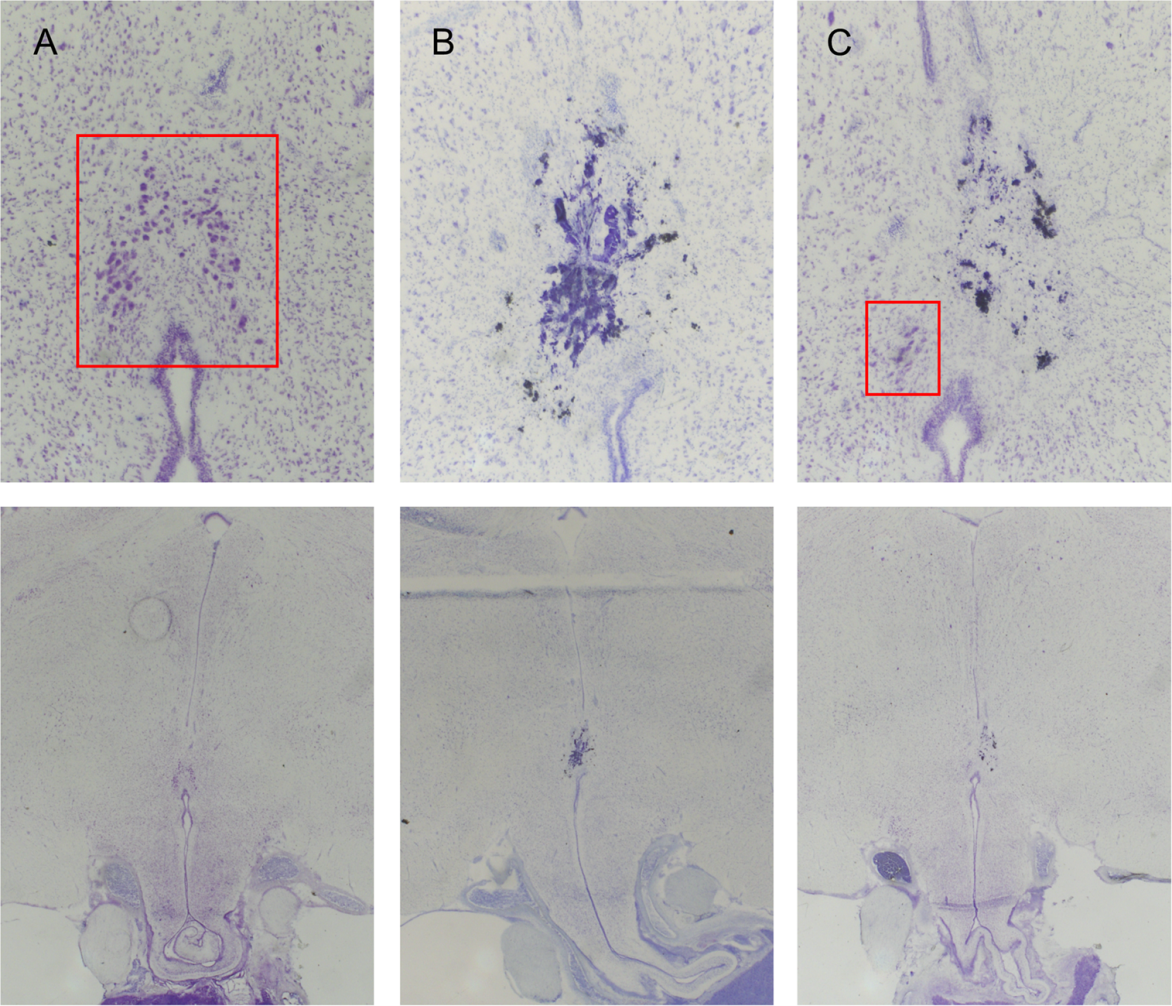
PMM lesions. Thionin-stained coronal sections of the hypothalamus show the PMM area of representative sham (A), lesion (B), and partial lesion (C) birds. The top panels (438x total magnification) show PMM area detail, while the bottom panels (73x total magnification) show general location within the caudal hypothalamus. In the top panels, the red squares demarcate an intact PMM (A) and the remaining PMM cells resulting from a partial lesion (C). The darkly stained material in (B) and (C) is damaged tissue resulting from the lesion.

For the S group (N = 42), 11 birds were determined to have full lesions of the PMM, 19 birds had partial lesions, and 6 birds were classified as missed lesions. Sham surgeries were performed in 6 birds. For the U group (N = 40), 9 birds had full PMM lesions, 22 birds had partial lesions, and 4 birds had missed lesions. Sham surgeries were performed in 5 birds. Examples of full, partial, and sham lesions are shown in Fig 1.

### Lay latency

**Fig 2:**
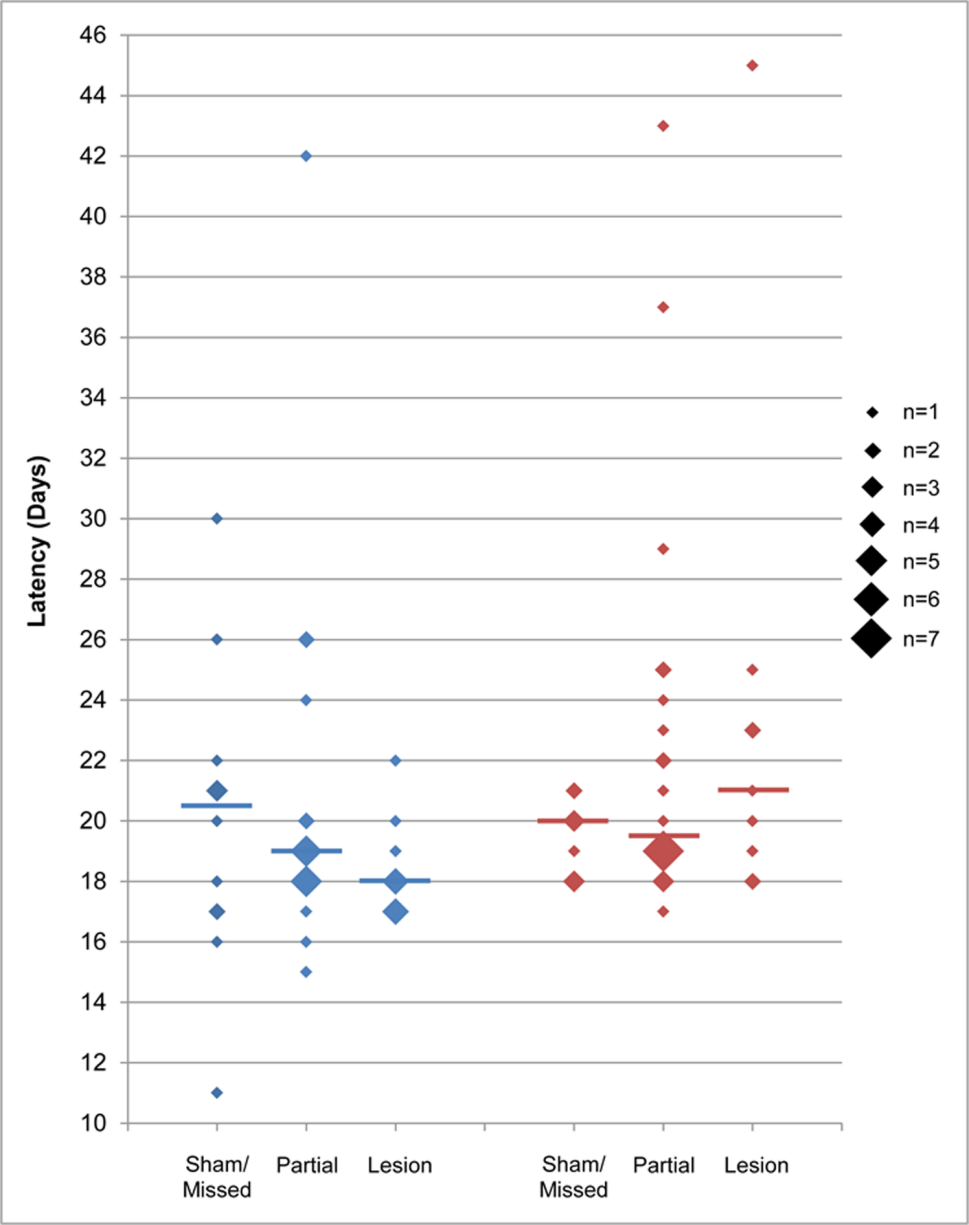
Lay latency is not affected by PMM lesion. Plots show the lay latency in days for each individual (diamonds). In cases where more than one individual had the same latency, the number of individuals is indicated by the size of the diamond. Group medians are indicated by horizontal lines. S group = blue; U group = red.

The median lay latency was 19 days, which is typical of domestic turkeys photostimulated at the age we used (median = 203 days) [32]. Fig 2 shows the latency data for individual birds, as well as the group medians. We did not find any evidence that PMM lesions affected lay latency. Lesion category group medians did not significantly differ from each other (Kruskal-Wallis; S group *p* = 0.36, U group *p* = 0.39). Furthermore, according to the regression analysis (Table 1), lesion category was not a significant predictor of lay latency, and the predicted model estimates for full and partial lesions did not differ from shams/missed lesions.

**Table 1:** Negative binomial regression analysis of lay latency as a function of lesion category.

Group		*df*	Estimate	SE	χ^2^	*P*
S	Intercept	1	2.996	0.067	1987.65	< .0001
	Partial Lesion	1	0.029	0.085	0.11	0.738
	Full Lesion	1	-0.090	0.099	0.83	0.363
	Lesion Category	2			1.76	0.416
U	Intercept	1	2.968	0.089	1105.33	< .0001
	Partial Lesion	1	0.146	0.104	1.95	0.162
	Full Lesion	1	0.192	0.122	2.46	0.117
	Lesion Category	2			2.71	0.258

Regression coefficients are modeled as the log of the expected count (latency) as a function of predictor variables. Estimates for partial and full lesions represent the expected difference in log counts between these groups and the reference group (sham/missed lesions). The results from the Type III analysis of lesion category are also shown.

### Egg production

**Fig 3:**
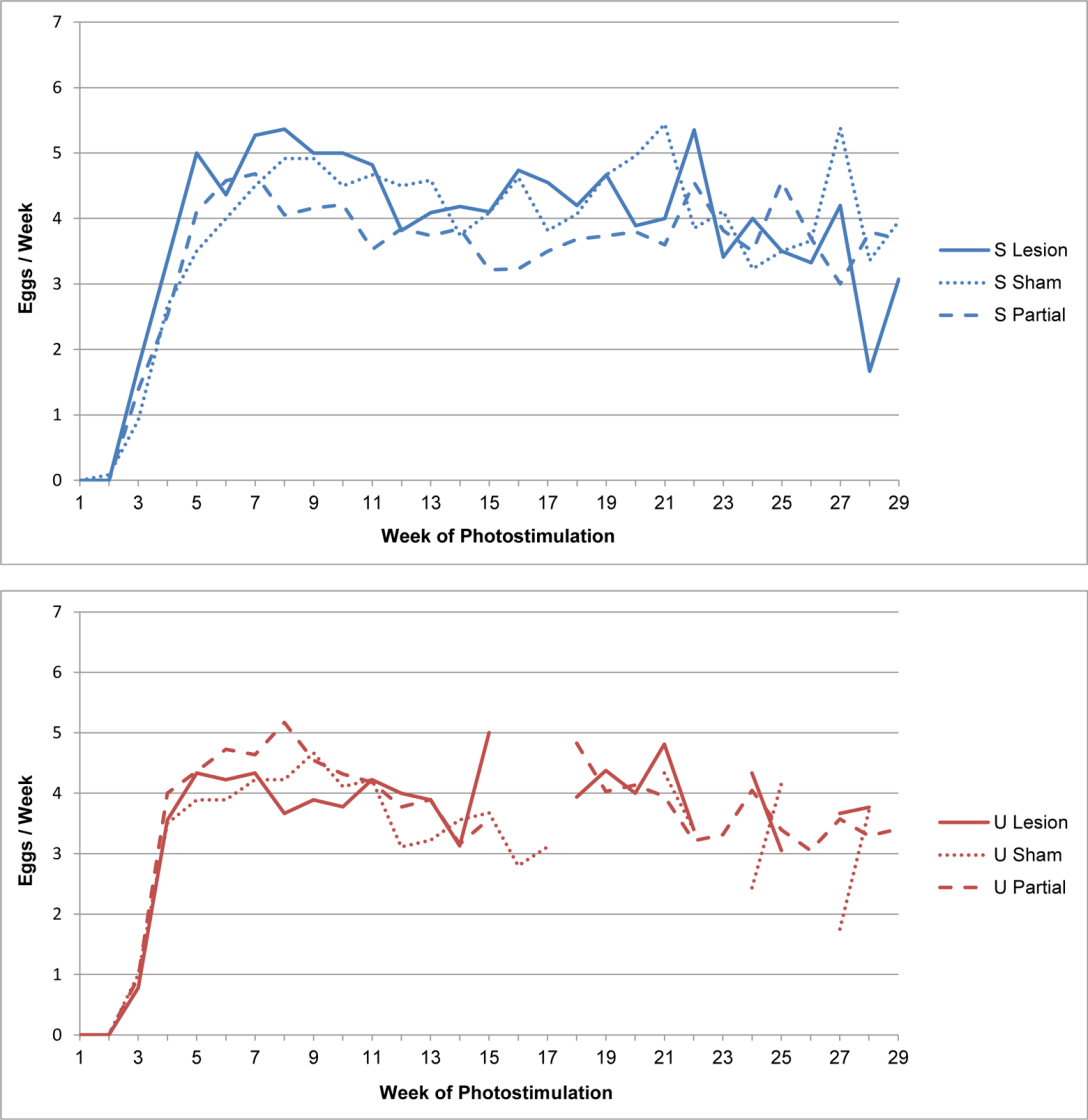
Egg production curves are not affected by PMM lesion. The mean weekly egg production for each group is plotted over time (in weeks). S group = blue; U group = red.

**Fig 4:**
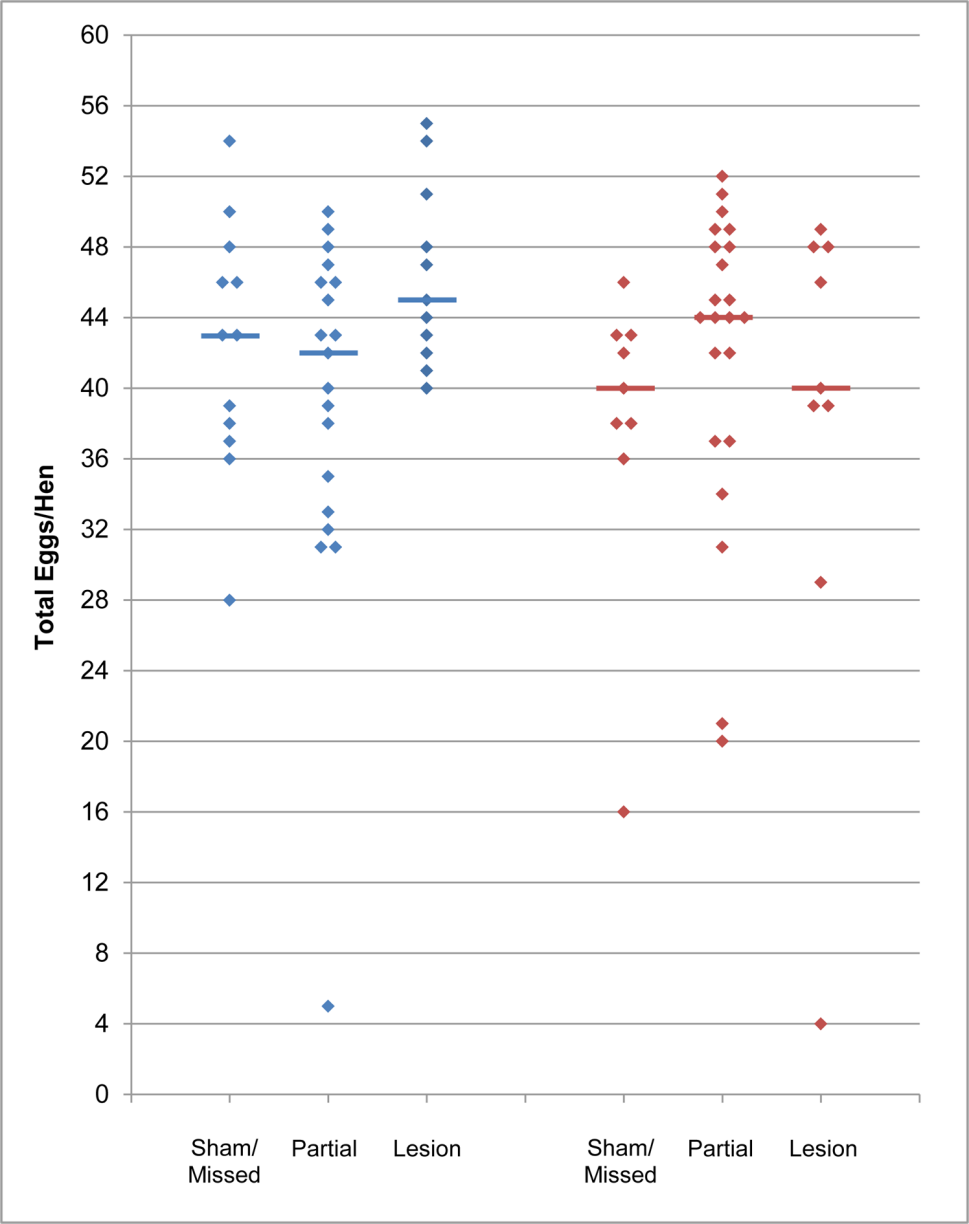
Total egg production is not affected by PMM lesion. Plots show the total number of eggs produced in the first 12 weeks following photostimulation for each individual (diamonds). Group medians are indicated by horizontal lines. S group = blue; U group = red.

**Fig 5:**
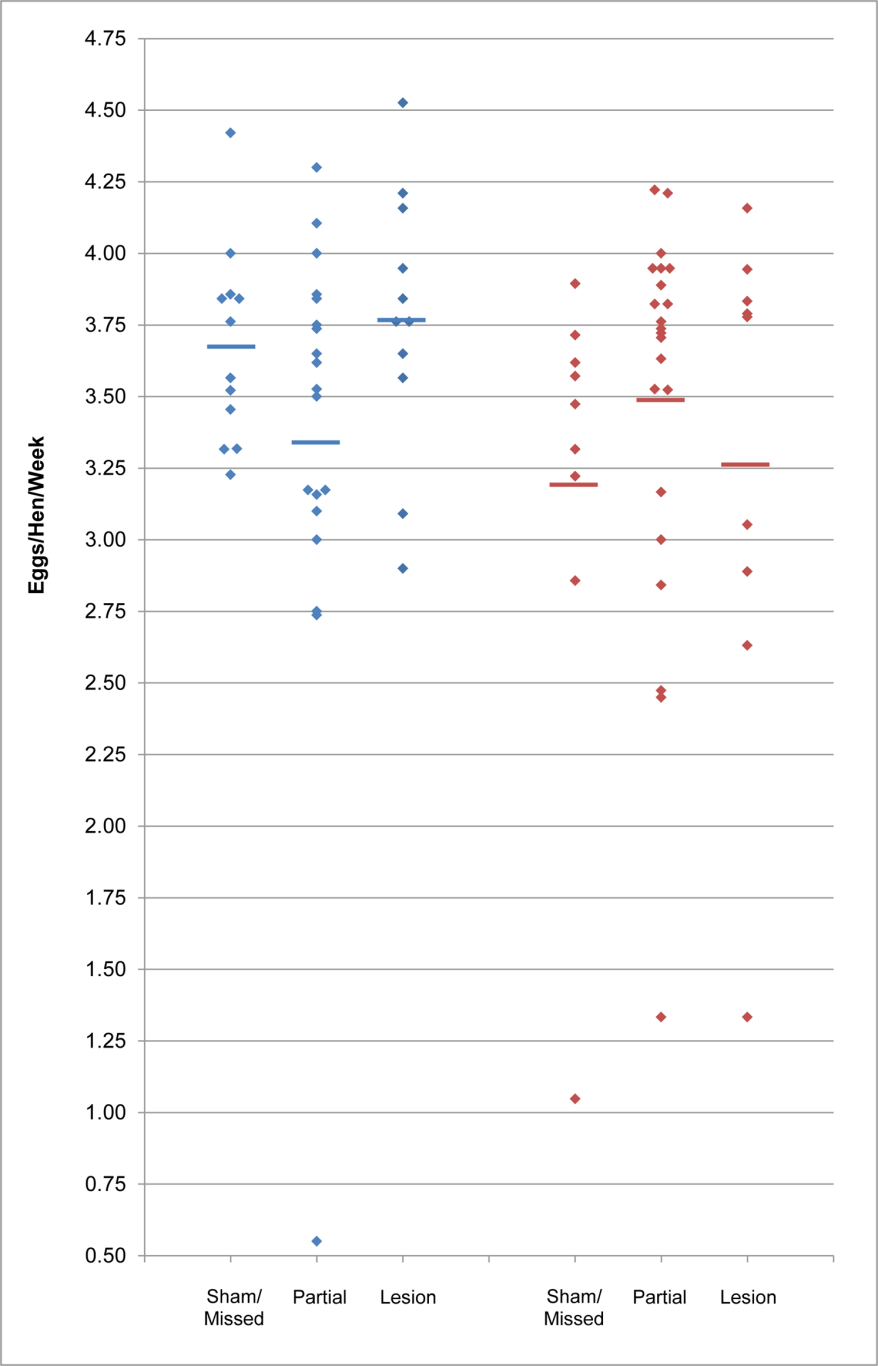
Weekly egg production is not affected by PMM lesion. Plots show the individual means (diamonds) for weekly egg production over the duration of the study. Group means are indicated by horizontal lines. S group = blue; U group = red.

Egg production curves for each group are shown in Fig 3, while Figs 4 and 5 show the total and mean weekly egg production data, respectively. Overall, peak, maintenance, and endpoint production are very similar to performance targets for the strain (Fig 3) [31]. We did not find any evidence that PMM lesions affected egg production. Lesion category group medians did not significantly differ for total egg production in the first 12 weeks (Kruskal-Wallis; S group *p* = 0.11, U group *p* = 0.23) nor for mean weekly production overall (Kruskal-Wallis; S group *p* = 0.19, U group *p* = 0.34). Moreover, for total12, regression analysis showed that lesion category was not a significant predictor of egg production, and the predicted model estimates for full and partial lesions did not differ from shams/missed lesions (Table 2). When analyzing egg production curves, we found a significant effect of time, but no significant effect of lesion category or the interaction of the main effects (Table 3A). These results indicate that egg production changed over time, as expected, but lesion categories did not differ from each other, nor did the change over time differ between lesion categories. Modeling time as polynomial did not change these results. Furthermore, the marginal means of full and partial lesion groups did not differ from sham/missed lesion groups (Table 3B).

**Table 2:** Negative binomial regression analysis of total egg production as a function of lesion category.

Group		*df*	Estimate	SE	χ^2^	*P*
S	Intercept	1	3.746	0.061	3736.89	< .0001
	Partial Lesion	1	-0.079	0.079	1.01	0.315
	Full Lesion	1	0.091	0.088	1.08	0.299
	Lesion Category	2			4.26	0.119
U	Intercept	1	3.638	0.098	1378.53	< .0001
	Partial Lesion	1	0.100	0.116	0.75	0.388
	Full Lesion	1	-0.000	0.022	0.00	1.000
	Lesion Category	2			1.14	0.565

Regression coefficients are modeled as the log of the expected count (total12) as a function of predictor variables. Estimates for partial and full lesions represent the expected difference in log counts between these groups and the reference group (sham/missed lesions). The results from the Type III analysis of lesion category are also shown.

**Table 3:** Hypothesis tests for main effects and differences between lesion categories following negative binomial regression analysis of weekly egg production over time.

**A.**	**Group**		**Num *df***	**Den *df***		***F***	***P***
	S	Lesion Category	2	213		0.31	0.734
		Time	1	267.5		101.07	< .0001
		Interaction	2	267.5		0.08	0.920
	U	Lesion Category	2	187.7		0.29	0.749
		Time	1	241		68.07	< .0001
		Interaction	2	241.1		0.25	0.778	
**B.**	**Group**		***df***	**Estimate**	**SE**	***t***	***P***
	S	Partial Lesion	159.8	-0.091	0.072	-1.27	0.205
		Full Lesion	161.6	0.026	0.079	0.33	0.742
	U	Partial Lesion	131.7	0.081	0.100	0.81	0.418
		Full Lesion	132.8	0.013	0.121	0.11	0.914

A) Hypothesis tests of fixed effects following negative binomial regression. B) Hypothesis tests for pairwise differences in the marginal means (log scale) for partial and full lesions compared to sham/missed lesions. Standard errors are adjusted for the covariance parameters included in the model.

### Gonadal regression

In the S group, 7 out of 42 birds had regressed gonads. The breakdown according to lesion category is as follows: full lesions, 1 out of 11; partial lesions, 3 out of 19; sham/missed lesions, 3 out of 12. In the U group, 9 out of 40 birds had regressed gonads. By lesion category, the numbers are: full, 1 out of 9; partial, 4 out of 22; sham/missed, 4 out of 9. Fisher’s exact test for a 2X3 contingency table revealed that lesion categories do not differ in the proportion of regressed gonads (S group *p* = 0.67; U group *p* = 0.30).

## Discussion

The photoperiodic breeding cycle can be separated into 3 active stages: the onset of breeding (photoinduction), the maintenance of breeding condition, and the termination of breeding (photorefractoriness). Our study clearly demonstrates that the PMM is not required for photostimulation or maintenance of the reproductive system in turkey hens. Bilateral ablation of the PMM did not alter the onset of lay following photostimulation, nor subsequent egg production under photostimulating conditions. Moreover, it appears that the PMM is not required for photorefractoriness, as indicated by lesioned birds with regressed gonads under photostimulatory conditions. These results contrast with a body of literature suggesting an important role of the PMM in photoperiodic timekeeping [28, 23, 26]. It should be noted, however, that previous studies did not directly evaluate the effects of PMM lesion on reproductive activity. While the PMM may contribute in some way to the photoperiodic system, our results indicate that it is not essential for regulating gonadal growth and reproduction. It is worth pointing out that electrolytic lesions can destroy not only cell bodies, but can also damage nearby fibers of passage. This can pose a problem of interpretation when an effect of the lesion is observed; in these cases, chemical lesions that target neurons can be used to clarify a structure's contribution. In our study, whether or not there was damage to fibers of passage, PMM lesions had no effect on any of the variables measured. The most parsimonious explanation is that the PMM does not play a role in regulating photoinduction of the reproductive system. We favor the more conservative conclusion that the PMM is not necessary for photostimulation. If the PMM does indeed play a role in photoperiodic regulation, its role must be redundant with other photoreceptive sites in the avian brain.

Our study included two different photostimulation treatments, designed to reveal potential roles of the PMM at different stages of the breeding cycle. In the S group, birds were photostimulated prior to PMM lesions. Thus, any effect of PMM lesions in this group would indicate that the PMM contributes to the maintenance of reproductive activity under long-day conditions after normal photoinduction has already occurred. Egg laying onset aligned with expected latency values [32], indicating that photoinduction did indeed occur as normally expected. After surgery, egg production in fully lesioned birds reached similar peak levels as compared to shams and missed lesions, was maintained at a similar production rate, and declined at a similar rate. This pattern of egg production demonstrates that the PMM is not essential for maintaining breeding state in photostimulated turkeys.

In the U group, birds received PMM lesions prior to photostimulation. This allowed us to investigate the role of the PMM in photostimulation itself. Surprisingly, the onset of lay appeared to occur normally in this group as well. As with the S group, production latency was within the normal range, and PMM lesion had no effect on latency to lay. These results clearly demonstrate that the PMM is not required for early key events during photostimulation: perceiving the photoperiodic light signal, measuring the photoperiod with a circadian clock, or the activation of output signals that stimulate the HPG axis, despite evidence that the turkey PMM contains the machinery for each of these functions [28, 23]. Additionally, we found no differences among lesion categories in peak production rates, maintenance production rates, or the gradual rate of decline of production, confirming results from the S group that the PMM is not essential for the maintenance of egg-laying following normal photostimulation.

Photostimulation of the HPG axis has been well studied, and the chain of events beginning with long-day induction of TSH in the PT, and culminating in release of gonadotropins from the pituitary and gonadal growth, has been described in detail [12, 33, 34]. The pathways by which the long-day stimulus is conveyed to the PT remain elusive, but they are known to require a hypothalamic circadian clock and deep-brain photoreceptors responsive to long days [2]. Our results clearly show that the PMM is not an essential component of this photoperiodic sensory system. As the breeding cycle progresses, continued egg production is dependent on the release of GnRH-1 at the ME and the resulting high circulating levels of plasma LH [12]. Precisely how the brain maintains GnRH-1 secretion is unknown, but inputs from either photoperiodic photoreceptors and/or photoperiodic circadian clocks are likely to be involved. Although it is clear that the PMM is not required for these functions, it may contribute as part of a redundant system. In addition to the PMM, birds are thought to possess photoreceptive neurons in the LSO, PVN, and PVO (reviewed in [16]). Each of these sites might play a specialized role in photoperiodic regulation, or they may represent multiple redundant sites of photoperiodic photoreception. Moreover, there may be species differences in the role of each site. In any given species, activation of one or more of these areas may be sufficient to induce activity within the HPG axis. Alternatively, one of these areas could be the master regulator on its own. Incidentally, we noted that one bird in the S group sustained complete ablation of the PVO, in addition to a full lesion of the PMM. Egg production appears normal in this bird (total12 = 45, eggs/wk = 3.8). This result suggests that the PVO is not essential for the maintenance of egg production following normal photostimulation in turkeys. No birds in the U group sustained complete ablation of the PVO, so we are unable to comment on the role of the PVO in photostimulation.

Although the precise neuroendocrine mechanisms are not well-understood, the termination of breeding and photorefractoriness are associated with decreased secretion of GnRH-1 and a reduction in circulating LH. In many songbird species, GnRH-1 protein content in the hypothalamus changes dramatically in response to photoperiod, and these changes are driven by photoperiodic regulation of *GnRH-1* transcription [35]. Thus, it is thought that a decline in GnRH-1 synthesis is a key event in the onset of photorefractoriness. In other species, such as Japanese quail, photoperiodic regulation appears to occur only at the level of GnRH-1 secretion, not availability [36]. The species differences in photoperiodic regulation of GnRH-1 are associated with variation along a continuum of photorefractory responses, with "absolute photorefractoriness" at one end and "relative photorefractoriness" at the other. In species with absolute photorefractoriness, such as European starlings (*Sturnus vulgaris*), gonads spontaneously regress under stimulating photoperiods, and do not recrudesce even if the photoperiod is lengthened. In species with relative photorefractoriness, such as Japanese quail, declining photoperiod initiates gonadal regression; increasing the photoperiod causes the gonads to recrudesce. Domestic turkeys are thought to fall somewhere in between these two extremes, showing large individual variation in the expression of relative and absolute photorefractoriness [37, 38]. Although some studies in turkeys have shown changes in *GnRH-1* mRNA following photostimulation [39, 25, 26], the importance of GnRH-1 synthesis in photoperiodic regulation in turkeys remains unclear. A recent study in turkeys raises the hypothesis that photorefractoriness is the result of increased GABAergic inhibitory activity in the PMM, resulting in the loss of photoperiodic entrainment of circadian oscillators thought to mediate the neuroendocrine response to the long-day stimulus [26]. Regardless of the mechanism, two lines of evidence in the current study suggest that the PMM does not play an essential role in photorefractoriness. First, egg production in all surgical groups declined from peak levels with a similar rate, suggesting that there were no detectable differences in the onset of the termination of breeding. Second, two animals with full lesions (one in the S group and one in U group) had regressed gonads at the end of the study, indicating that the PMM is not necessary for photorefractoriness, and there was no significant difference among lesion categories in gonadal regression. However, across all groups, the incidence of gonadal regression was rather low. Although we may be tempted to speculate that the PMM plays no direct role in regulating photorefractoriness, we cannot draw any definitive conclusions, as our study did not directly address the effects of PMM lesions on photorefractoriness. Future studies focusing specifically on the development of photorefractoriness in PMM-lesioned birds, including the manipulation of photo-regimes to reveal absolute vs. relative photorefractoriness, would more clearly elucidate any role of the PMM in photorefractoriness, if it indeed exists.
